# Co-Effects of Nitrogen Fertilizer and Straw-Decomposing Microbial Inoculant on Decomposition and Transformation of Field Composted Wheat Straw

**DOI:** 10.3390/life13101993

**Published:** 2023-09-29

**Authors:** Hiba Shaghaleh, Yuanpeng Zhu, Xinyi Shi, Yousef Alhaj Hamoud, Chao Ma

**Affiliations:** 1 Anhui Province Key Laboratory of Farmland Ecological Conservation and Pollution Prevention, Research Centre of Phosphorus Efficient Utilization and Water Environment Protection along the Yangtze River Economic Belt, College of Resources and Environment, Anhui Agricultural University, Hefei 230036, China; hiba-shaghaleh@njfu.edu.cn (H.S.);; 2 College of Environment, Hohai University, Nanjing 210098, China; 3 Key Laboratory of Water Cycle and Related Land Surface Processes, Institute of Geographic Sciences and Natural Resource Research, Chinese Academy of Sciences, Beijing 100101, China; 4 College of Hydrology and Water Resources, Hohai University, Nanjing 210098, China

**Keywords:** field composting, straw decomposition and transformation, co-effect, nitrogen fertilizer, straw-decomposing microbial inoculant

## Abstract

Although straw is an abundant and useful agricultural byproduct, it, however, exhibits hardly any decomposition and transformation. Despite the successful application of chemical and biological substrates for accelerating straw decomposition, the co-effects and mechanisms involved are still unknown. Herein, we performed a 120 day field trial to examine the co-effects of a nitrogen fertilizer (N) and a straw-decomposing microbial inoculant (SDMI) on the straw mass, nutrient release, and the straw chemical structure of composted wheat straw in the Chaohu Lake area, East China. For this purpose, four treatments were selected with straw: S (straw only), NS (N + straw), MS (SDMI + straw), and NMS (N + SDMI + straw). Our results indicated that NMS caused a higher straw decomposition rate than S, NS, and MS (*p* < 0.05) after 120 days of composting. The N, P, and K discharge rates in treating with NMS were higher than other the treatments at 120 days. The A/OA ratios of the straw residues were gradually increased during the composting, but the treatment of NMS and MS was lower than the CK at the latter stage. The RDA showed that the decomposition rate, nutrient release, and the chemical structure change in the straw were cumulative, while respiration was strongly correlated with lignin peroxidase, manganese peroxidase, and neutral xylanase. In conclusion, nitrogen fertilizer or straw-decomposing microbial inoculant application can improve the decomposition rate and nutrient release with oxidase activity intensified. However, the co-application of nitrogen fertilizer and a straw-decomposing microbial inoculant promoted straw decomposition and enzyme activity better than a single application and showed a lower decomposition degree, which means more potential for further decomposing after 120 days.

## 1. Introduction

Crop production unavoidably results in large amounts of straw residues. However, a minor amount of the straw produced is employed in soil improvement and bioenergy [1,2], while a major amount of the straw is discarded to prepare the land for the next crop season [3]. Returning the crop straw to the field is suggested to increase the soil’s organic carbon storage [4,5]. Also, burying straw in soil has possibly encouraging effects on soil management [6] as it could improve both the soil fertility and it’s biological activities, finally increasing the sustainability of cropping systems [7,8]. Therefore, recent studies were conducted to promote the efficiency of straw degeneration [9,10].

Lignocellulose is a major constituent of crop straw, which includes cellulose (35–50%), hemicellulose (20–30%), acid-soluble lignin, and acid-insoluble lignin (20–30%) [11,12]. Although lignocellulose could be a valuable source of soil organic matter [13,14], it is enclosed within a recalcitrant and hardly decomposable lignin. Farmers were recently encouraged to utilize straw for either energy, substrate, material, feed, or for fertilization. Therefore, most farmers have preferred to put the straw in their fields to decompose naturally because of the high transportation costs and the disadvantages of returning the straw directly. However, implementing straw composting is still difficult due to its slow decomposition rate and the complex and changeable field environmental conditions, causing uncertainty in straw decomposition progress, with most composting products being useless [15]. Numerous studies have been carried out to promote the efficiency of straw composting. However, most of these studies have been done on a pilot scale, and studies on the field conditions are still lacking; thus, detecting the efficiency of straw degeneration in field conditions is of great interest.

Straw decomposition involves physical, chemical, and biological responses that might be affected by external factors/additives [16,17]. Jiang (2018) reported that adding nitrogenous substrates (N) during reduced composting nitrogen (N) loss stimulated the decomposition degree [18]. Also, it was found that straw-decomposing microbial inoculants (SDMIs) could accelerate the straw decomposition rate, increasing nutrient release; thus, SDMIs were suggested as an eco-friendly way for crop growing [19]. Studies involving exogenous microbes (i.e., SDMI) or nitrogen fertilizers (i.e., urea), focused on single-factor research. Moreover, studies combining inoculants with a nitrogen fertilizer and an artificial manure, and reflecting on their relationship were very few, particularly on a field level [20,21]. Both N and SDMIs have a great impact on the decomposition of straw. Additionally, adding an appropriate amount of inorganic nitrogen benefits straw decay [22]. However, high inorganic nitrogen levels can constrain microbial activity, affecting straw degeneration [23]. Previous studies proposed that microorganisms play a fundamental part in straw degeneration during composting. Meanwhile, applying straw-decomposing microbial inoculants can promote straw decay considerably [19]. Therefore, the impact and mechanism of incorporating both N and SDMIs on straw degeneration under field conditions is still largely uncertain.

Notably, the previous studies have only focused on straw mass loss using a single-factor analysis; however, nutrient release is a major indicator for assessing straw decomposition [24,25]. Moreover, the chemical structure transformation is an additional index to evaluate the degree of straw degeneration that is easier to describe than both the mass loss and nutrient discharge [21]. Baddi et al. (2004) effectively applied the Fourier transform infrared spectrometer (FTIR) and Carbon-13 nuclear magnetic resonance spectrometry (^13^C-NMR) to identify the content of fulvic acid in olive mill waste and grain straw under composting conditions to estimate the final product’s maturity [26]. Using a two-month composting trial, Blanco and Almendros (1997) explored the chemical maturity of wheat straw via ^13^C-NMR [27]. To date, most researchers have used advanced detection techniques to determine a fixed phase of the entire composting process [15,28]. However, the dynamics of straw transformation through composting has seldom been observed.

Microbes play a central part in straw degeneration during composting since the exogenous microbes can promote cellulose decomposition by either motivating enzyme activity or modifying the microbial community [19]. Many studies suggested the use of different procedures to promote straw degradation in composting. Zhou (2015) promoted bacteria communities’ richness and diversity by changing the condition of the compost temperature [28]. Zhang and Marschner (2017) and Xu et al. (2019) stimulated the microbial community and enzyme activity by regulating the straw water content and combining different types of straw [20,29]. Moreover, the combination of an SDMI and N fertilizer greatly impacts the decomposition of straw. Adding an appropriate amount of inorganic N is beneficial for straw decay, while high levels of inorganic N will constrain the microbial activity affecting straw degeneration [23]. Previous studies have proposed that microorganisms play a fundamental part in straw degeneration during composting. However, the straw compost was only carried out using a single factor, for example either the N fertilizer or the straw-decomposing microbial inoculant. Furthermore, relationships among N, SDMIs, enzyme activity, and straw decomposition indices (mass, nutrient, and chemical structure) remain unclear. We hypothesized that the co-application of N and an SDMI increases the enzyme activity, which, in turn, affects straw mass loss, straw chemical structure/composition, and nutrient release. Therefore, this study aimed to elucidate on the in situ estimation of the decomposition rate, nutrient release, and chemical structure change in the straw during straw composting with the addition of N and SDMI; and on the key enzymes (cellulase or ligninase) and the main mechanisms they use for straw degradation.

## 2. Materials and Methods

### 2.1. Experimental Site Description

The straw composting field experiment was conducted in Wanzhong Comprehensive Experimental Station of Anhui Agricultural University, Lujiang County, Anhui Province (31°25′ N, 117°09′ E). In the study area, and during the experimental period, the mean temperature was 16.5 °C, the maximum temperature was 40 °C, the minimum temperature was −7 °C, and the average precipitation was 1000 mm. The soil was classified as paddy soil, and the cropping approach was wheat-rice rotation; thus, the leading straws were wheat straw and rice straw. The soil physiochemical properties before the experiment were as follows: clay 19.96%, silt 39.85%, sand 40.19%, and soil total organic carbon 16.39 g·kg^−1^.

### 2.2. Material Preparation, Experimental Design, and Treatment Application

The straw-decomposing microbial inoculant (SDMI) was obtained from the Institute of Agricultural Resources and Regional Planning, Chinese Academy of Agricultural Sciences, Anhui, China. The SDMI contained *Bacillus subtilis* (2.21 × 108 cells·g^−1^), *Ballicus stearothermophilus* (0.0054 × 108 cells·g^−1^), *Sreptomyces colin* (0.17 × 108 cells·g^−1^), and *Streptomyces leucocephala* (2.76 × 108 cells·g^−1^). The composting straw (<10 cm) was collected from the nearby fields after the wheat harvest, which contained organic carbon 40.42%, total nitrogen 0.82%, total phosphorus 0.10%, and total potassium 1.30%. The chemical composition of the straw was evaluated by applying the analytical procedure suggested by the National Renewable Energy Laboratory [30]. In this study, the experiment was conducted in a split-block design. Four treatments with three replications were implemented, including straw natural composting (S), straw composting with nitrogen application (NS), straw composting with an SDMI application (MS), and straw composting with nitrogen and an SDMI co-application (NMS). Therefore, twelve experimental plots with 6 m^3^ (3 m length × 2 m width × 1 m depth) were established under open field conditions. For all treatments, nitrogen (urea) and an SDMI were applied equally at the rates of 7.70 and 1.40 kg·plot^−1^, respectively, which is proposed as the standard quantity supplied [9]. Five nylon mesh bags (aperture 0.15 mm; 30 cm length; 50 cm width) filled with 20 g of straw were placed at 0.5 m depths in each plot. Three nylon mesh bags for each treatment were selected randomly and destructively sampled at 7, 15, 30, 70, and 120 days after composting for the determinations of straw mass, nutrient concentration, chemical composition, and lignocellulose activity.

### 2.3. Measurements of Straw Decomposition and Nutrient Release

The collected nylon mesh bags were transferred to the laboratory, and the straw was thoroughly washed with deionized water and oven-dried (75 °C) to a constant weight. Finally, the rate of straw decomposition was calculated using Equation (1) as follows:

(1) $$R_{s}=\frac{\left(M_{0}-M_{t}\right)}{M_{0}}\times 100\%$$

where R_s_ is the rate of straw decomposition (%), M_0_ is the initial straw mass (g), and M_t_ is the straw mass (g) after the days of composting.

The standard methods determined the basic nutrient properties of the straw samples. The air-dried straws were sieved (<2 mm), digested using H_2_SO_4_ and H_2_O_2_ at 400 °C in a graphite oven for 120 min, and then stored in 100 mL glass cylinders for analyses. The total N was detected using the Kjeldahl protocol [31]; the total P and total K were measured using the molybdenum blue protocol and flame photometry, respectively [32].

### 2.4. Straw Chemical Analyses

The ^13^C multi−CP NMR spectra were obtained using a Bruker AVANCE Ⅱ 400 spectrometer (Bruker Instruments, Billerica, MA, USA) operating at 100 MHz (400-MHz ^1^H frequency). All the experiments were run in a double-resonance probe head using 4 mm sample rotors. Multiple cross-polarization (^13^C multi−CP) NMR experiments were obtained with good sensitively using a spinning speed of 14 kHz, a recycle delay of 0.25 s, a 90° pulse for ^1^H, 4 μs 90° pulse for ^13^C, and a cross-polarization time of 1.5 ms. Sub-spectra for non-protonated and mobile carbon groups were obtained by combining the ^13^C multi−CP sequence with 68-μs dipolar dephasing (multi−CP/DD). The scans for the straw samples’ multi−CP/DD experiments were 12,228. The total signal intensity and the proportion contributed by the C functional groups were determined by the integration of the spectra in the chemical-shift area [33] 44–0 ppm (Alkyl C), 64–44 ppm (OCH3), 93–64 ppm (O-alkyl C), 113–93 ppm (Anomeric C), 142–113 ppm (Aromatic C-C), 162–142 ppm (Aromatic C-O), 188–162 ppm (COO/N-C=O), and 220–188 ppm (C=O). The proportion ratios of alkyl C to O-alkyl C (A/OA) were applied to assess the organic matter’s straw decomposition degree and humification.

### 2.5. Determination of Enzyme Activities

The activity of cellulase, xylanase, manganese peroxidase (MnP), lignin peroxidase (LiP), and laccase (Lac) was determined using an analysis kit (Suzhou Branch Ming Biotechnology Co., Ltd., Beijing, China). The xylanase was used to analyze the hemicellulose degradation via the straw xylan as the substrate. The enzyme activity was counted by each μmol of glucose or xylose per minute produced, and this was regarded as the cellulase or xylanase activity. The MnP, LiP, and laccase were used to measure the lignin degradation. The activity of these enzymes were detected through veratryl alcohol, Mn_S_O_4,_ and ABTS as substrates, respectively [34].

### 2.6. Statistical Examination

A one-way analysis of variance (ANOVA) was applied to assess the effects of the different treatments on straw decomposition, nutrient releases, and enzyme activities. The Duncan’s multiple range test was employed to identify the significance of variations among the treatments at *p* ≤ 0.05. A redundancy analysis was employed to reveal the relationship between the enzyme activity and the decomposition rate of the straw. All the statistical analyses were implemented using SPSS 22.0 (IBM Co., Armonk, NY, USA).

## 3. Results

### 3.1. The Effects of Nitrogen Fertilizer and Microorganisms on the Straw Mass and Nutrient Release

**Figure 1:**
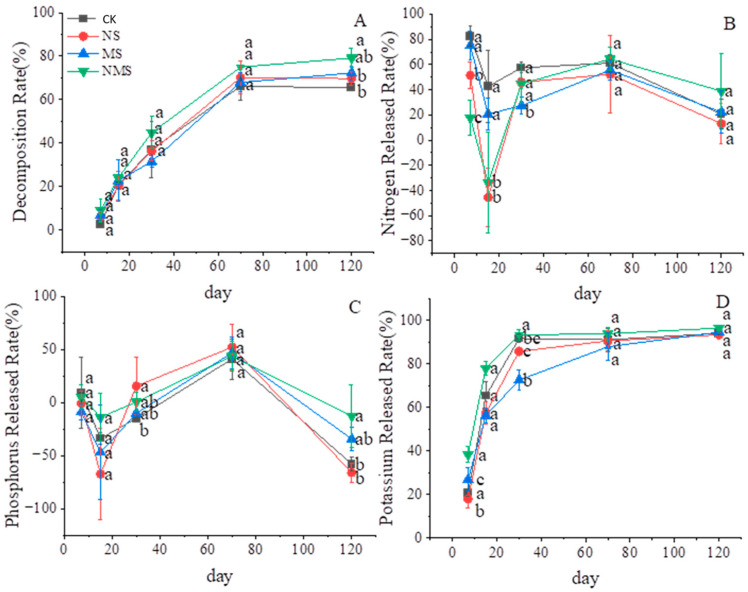
Patterns of the decomposition rate of wheat straw (**A**); the released rate of different nutrients (**B**–**D**); CK, straw natural composting; NS, straw compositing with nitrogen application; MS, straw compositing with SDMI application; NMS, straw compositing with nitrogen and SDMI co-application. Each bar is the standard deviation of three replicates. Numbers followed by different lowercase letters in the same row are significantly different at *p* > 0.05.

The changes in straw decomposition under different treatments and stages of compositing are shown in Figure 1A. Overall, the NMS treatment considerably decomposed 79% of the straw after composting (*p* < 0.05). Whereas the other treatments caused a similar straw decomposition rate of about 70%. Furthermore, the highest and the lowest rates of straw decomposition were observed at 120 days and 7 days, respectively, with mean values of 71.64% and 6.21%, suggesting an increase in the decomposition rate over the decomposition time.

The rates of nutrient release from the straw are presented in Figure 1B–D. During the straw decomposition process, the K release rate increased gradually, showing a trend of rapid release at the first stage and a slow-release trend during the latter stage. However, the changes in the release rate of N and P are similar, decreasing at the first stage (15–30 days), then gradually increasing, and declining at the latter stage (70–120 days). The N, P, and K discharge rates in treating with NMS were higher than the other treatments at 120 days. The K release rates of NMS were 114.13% and 43.12% higher than NS and MS at 7 days, respectively, and 1.77%, 8.78%, and 28.61% higher than S, NS, and MS at 30 days, respectively (*p* < 0.05).

### 3.2. The Effects of Nitrogen Fertilizer and Microorganisms on the Straw Chemical Composition

**Figure 2:**
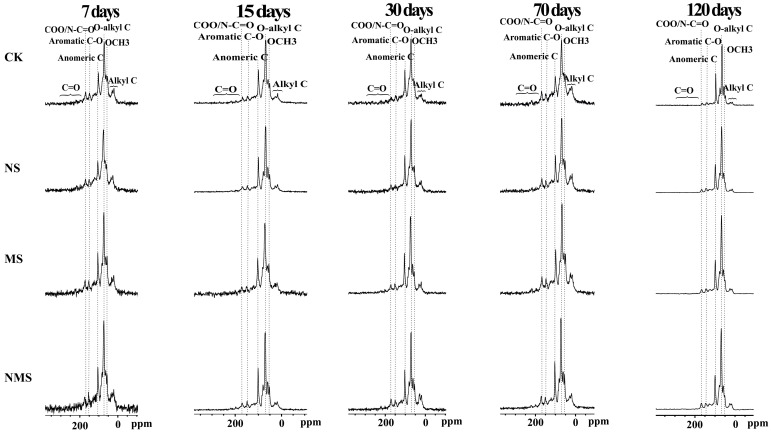
Illustrations of the relative proportions of C-containing functional groups in straw expressed as a percentage of the entire ^13^C spectrum are given in Table 1. CK, straw natural composting; NS, straw compositing with nitrogen application; MS, straw com-positing with SDMI application; NMS, straw compositing with nitrogen and SDMI co-application. In accordance, alkyl C (0−44 ppm) accounted for most of the total C (0−220 ppm), followed by O−alkyl C (64−93 ppm), aromatic C−C (113−142 ppm), NCH/OCH3 (44−64 ppm), COO/N−C=O (162−188 ppm), anomeric C (93−113 ppm), aromatic C−O (142−162 ppm), and C=O (188−220 ppm) (shown in Table 1).

Figure 2 shows the ^13^C multi-CP/MAS NMR and the corresponding dipolar-dephased spectra (DD) from all the treatments, which provides quantitative structural information. The comparative abundance of alkyl C (0−44 ppm) and aromatic C−C (113−142 ppm) all increased, but a reduction in the relative abundance of O–alkyl C (64−93 ppm) over decomposition time. The A/OA ratios of straw residue under NMS and MS conditions gradually increased from 7 to 70 days, slightly declining at 120 days. The A/OA ratios of straw residues under the NMS and MS conditions were higher than those under the CK and NS conditions from 7 days to 30 days, but it was lower than when under the CK condition at 70 days (Table 1).

### 3.3. The Effects of Nitrogen Fertilizer and Microorganisms on the Straw Enzyme Activity

**Figure 3:**
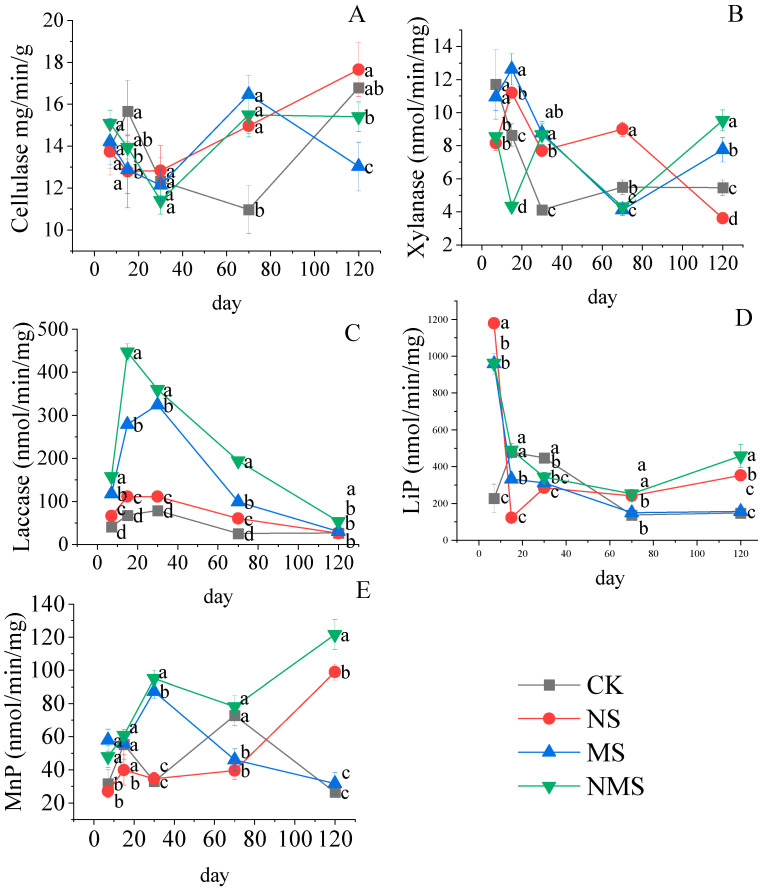
The dynamic changes of cellulose (**A**), xylanse (**B**), laccase (**C**), lignin peroxidase (LiP) (**D**) and MNP manganese peroxidase (MnP) (**E**) enzymes at different stages of straw decomposition. CK, straw natural composting; NS, straw compositing with nitrogen application; MS, straw com-positing with SDMI application; NMS, straw compositing with nitrogen and SDMI co-application. Each bar is the standard deviation of three replicates. Numbers followed by different lowercase letters in the same row are significantly different at *p* > 0.05.

The activity of straw lignocellulase in the different treatments is shown in Figure 3. Overall, the laccase activity showed a rapid increase at the early stage, followed by a gradual decrease at the latter stage. The lignin peroxidase activity showed a rapid decrease at the early stage, followed by a gradual increase at the latter stage. In contrast, the cellulase, xylanase, and manganese peroxidase activity showed insignificant dynamics during composting. The activity of cellulose, neutral xylanase, laccase, lignin peroxidase, and manganese peroxidase in the treatment with NMS were significantly higher than the other treatments at 120 days (*p* < 0.05). The laccase of the NMS had the highest activity at 15 days, which was 557.98%, 301.09%, and 60.39% higher than S, NS, and MS treatments, respectively (*p* < 0.05).

### 3.4. Relationship between Straw Decomposition and Straw Lignocellulase Activities

**Figure 4:**
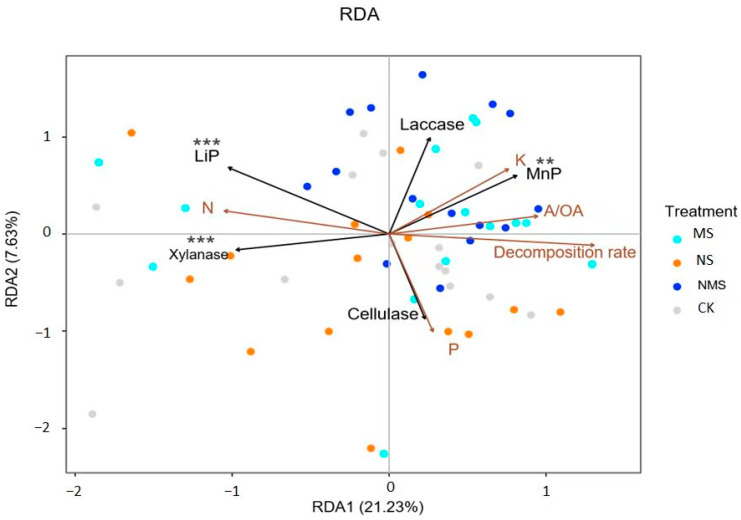
Redundancy analysis (RDA) of enzyme activity, the nutrient release rate and the decomposition rate of straw. CK, straw natural composting; NS, straw compositing with nitrogen application; MS, straw com-positing with SDMI application; NMS, straw compositing with nitrogen and SDMI co-application. LiP and MNP stands for lignin peroxidase and manganese peroxidase, respectively, ** and *** stands for *p* < 0.01 and *p* < 0.001, respectively.

The redundancy analysis (RDA) showed that the decomposition rate, nutrient release, and chemical structure change in the straw were cumulative, and respiration was strongly correlated with lignin peroxidase, manganese peroxidase, and neutral xylanase (Figure 4). The decomposition rate tended to be negatively correlated with manganese peroxidase but positively correlated with lignin peroxidase and neutral xylanase. The P and K release rates were positively correlated with manganese peroxidase but negatively correlated with neutral xylanase and lignin peroxidase. In contrast, the release rate of N was positively correlated with neutral xylanase and lignin peroxidase but negatively correlated with manganese peroxidase. The A/OA was negatively correlated with neutral xylanase and lignin peroxidase but positively correlated with manganese peroxidase.

## 4. Discussion

### 4.1. Nitrogen Fertilizer and Microorganisms Improved the Rate of straw Decomposition

In our field trial, the addition of the N fertilizer and microorganisms promoted straw mass loss and accelerated straw decomposition. This is consistent with the previous studies [12,35,36]. In a long-term straw-reforming study, Zhao and Zhang (2018) fertilization improved straw decomposition by increasing the bacterial and fungal biomasses [37]. However, our results showed that the addition of a nitrogen fertilizer inhibited the decomposition process at 120 days. Chen et al. (2018) found that nitrogen application increases the abundance of recalcitrant compounds in the soil organic matter [38]. Li et al. (2017) found that urea fertilization decreased the decomposition of the SOM and maize residues [23]. The reason might be the existence of a recalcitrant substance; it is hard to decompose these substrates in circumstances without an exogenous microbe. The results also showed that the decomposition rate constant (k) of MS is 0.019, far less than NS (0.024) or NMS (0.024). Zhang’s findings indicate that the soil organic matter (SOM) mineralization might be improved in N and P enriched ecosystems, with microbes being restricted due to the C limitation [29], which is consistent with our study. It might be that lower substrate concentrations inhibit the substrate catalysis by enzymes in microorganisms. It might also be that the cost of biochemical machinery limits the microbial decomposition of substrates at low concentrations [39]. This is enough to prove that crop and soil symbiotic systems in cultivar field conditions enable the decomposition microbes to obtain sufficient nutrients to finish the residual degradation process.

Nonetheless, an enclosed composting environment is averse to microbe’s competition for resources. Compared to NS and MS, NMS showed the benefits of a higher decomposition degree (mass loss) and a faster decomposition rate (k). Allison used a microcosm approach, proposing that decomposition rates should increase once the substrate concentrations are sufficient to overcome the biochemical investment costs [39]. Liu supported that the plant residue amendment to fertile soil is likely more effective for soil carbon accumulation and soil fertility buildup than infertile soil [40]. This is consistent with our results. Nitrogen provided the nutrients to prime effect at the initial stage of the straw decomposition, and microbes ensured the sustainability of the decomposition process at the latter stages [41,42,43]. NMS increased the decomposition rate, which might be due to the synergistic effect between these exogenous additions.

### 4.2. N Fertilizer and Microorganisms Enhance the Nutrient Release of Straw Decomposition

Many previous studies reported that the nutrients of the straw are released at different intervals during decomposition [44,45]. Moreover, they also pointed out that the nutrients are released at the latter stages with enrichment at the early stages. There were fewer studies about micronutrients in straw composting.

The result of the nutrients released in our study shows that straw nutrients are released quickly using the co-application treatment. However, a single application promoting straw nutrient release is insignificant compared to co-application (Figure 1B). In our research, microbial activity and nutrient deficiency limited soil organic matter decomposition. At the beginning of the decomposition, there is a rich source of N and P nutrients for strengthening the community of microbial decomposers; thus, the release rates of N and P nutrients reached the maximum rate in the first few days. After 20 days, the microbial activity is reduced by nutrient deficiency. Between 40 and 60 days, the release rates of N and P nutrients improved the process of microbial proliferation, which sharply decreased after 120 days. Our study reveals that the co-application of nitrogen and microbes provides the necessary nutrients for microbe succession and boosts the number of degradation bacteria. Analyzing the regulation of all the elements released, we found that some nutrients (N, P, Fe, Zn) manifest a phenomenon that is enrichment at an early stage, and the enrichment trend was amplified with the addition of nitrogen. Yin et al. (2021) found a similar manifestation [46]. The reason for the enrichment might relate to a priming effect, which happens in litter decomposition at the initial stage, which needs a proper C:N rate to finish the process of assimilation. In addition, different elements from straw significantly affect the decomposing environment. For example, nitrogen and phosphorus are essential nutrients for microbes to complete their life cycle and are consumed hugely during decomposition. Iron is usually considered a key agent in microbe transformation by providing an ion carrier and regulating the ambiance of composting by altering its pH. The plant is poisoned by zinc by transforming the root architecture, and the enrichment of zinc makes a hypotoxic environment for a microbe or plant development [47]. However, potassium and copper flowed away during composting, for they are the most liable and plentiful nutrients with an ionic state during composting. In addition, copper is supposed to be the coordination ion of laccase and cooperates with manganese in the process of decomposition enzyme synthesis.

### 4.3. Nitrogen Fertilizer and Microorganisms Improved the Activity of the Stalk Rot Enzyme

In this study, we found that a single application of an exogenous factor (e.g., N or microorganisms) caused an insignificant increase in enzyme activity, meaning that the increased enzyme activity was short-term and cannot be sustained during the entire decomposition process (Table 1). Similarly, some other studies observed a limit in the decomposition enzyme activity in the composting process. It may result from a low demand for nutrients by soil microorganisms in the presence of available nutrients. Generally, extracellular enzyme activity, that is, β-glucosidase, α-glucosidase, cellobiohydrolase, and β-xylosidase, depend on nutrient availability. The co-application of the microorganisms and N improved enzyme activity (laccase, lignin peroxidase, and Mn−dependent peroxidase) during the whole period of straw decomposition, and our results indicated that the co-application of microorganism and N caused higher oxidase activity when compared with the single application of these factors. Likewise, the previous studies concluded that oxidase activity improved with nutrient addition [48]. It might be that the balance between nutrients and bacteria causes a negative feedback mechanism. Meanwhile, the mechanism of nutrient control on enzyme activity is related to microbial metabolism’s economic theory, which predicts that enzyme activity production declines in the presence of simple nutrients while increases when complex nutrients are abundant. Besides, it is hard for microorganisms to colonize a new environment when competition happens between the exogenous and indigenous microorganisms if nutrients are available in insufficient amounts [49,50].

### 4.4. The Positive Relationships of Straw Decomposition and Enzyme Activities with Nutrient Addition

Generally, nutrient addition plays a pivotal role in straw decomposition and formation by regulating enzyme activity. In this study, specific enzyme taxa were strongly correlated with various nutrient additions. Consequently, linking straw decomposition and enzyme activity with nutrient addition is necessary.

To identify the links described above, we established a set of models using SEM that assessed the relationships between nutrient addition, various enzyme activity, and the decomposition indices (mass loss, nutrient released, and chemical structure). The SEM suggested that the addition of microbes was negatively correlated with the straw decomposition rate, while nitrogen addition showed the opposite result. Nitrogen improved the rate by increasing the laccase activity (Figure 3A). Bei et al. (2018) found that the soil and fungal communities were significantly increased with chemical fertilizer and manure [51]. Moreover, the present study is consistent with the previous studies. Burke et al. (2011) also reported that fungal communities were positively correlated with soil enzymes involved in carbon C, N, and P cycling [52]. The SEM revealed that nitrogen or the addition of microbe boosted the nutrients released, while the addition of nitrogen showed a significantly negative correlation with the nitrogen release.

Meanwhile, nitrogen or the addition of microbes were strongly correlated with oxidase (laccase and MnP) activity (Figure 3B). In previous studies [53], exogenous nutrient addition promoted straw nutrient release by raising the activity of hydrolases or oxidases, which is consistent with our studies. Furthermore, we found enzyme activity did not significantly relate to nutrient release. Microbes might absorb nutrients and enrich them through assimilation [54]. Straw decomposition decreased bacterial richness by increasing the abundance of specific taxa even though the nutrient component content increased [55]. The SEM showed that nitrogen and the addition of microbes were significantly positively correlated with MnP and laccase, and MnP and laccase were significantly correlated with A/OA (Figure 3C). This suggests that nitrogen and microbes, as the most available exogenous nutritious component, can enhance microbial activity, promote enzyme activity, and, thus, promote straw transformation. The present study is consistent with the previous studies [38,56].

Moreover, Feng et al. (2019) found that the laccase enzyme is the main factor in lignin accumulation in straw incorporation. The N availability or some specific bacteria communities were also strong factors [57]. The A/OA, as a vital index, elucidates upon the degree of decomposition [58]. We found that MS exhibited greater alkyl C and lower O-alkyl and aromatic C compared to NS, suggesting a greater degree of decomposition of organic matter in MS than in NS, and a previous study reached the same conclusion [59].

## 5. Conclusions

The straw compost integrated with nitrogen and microbes decomposed faster due to the higher enzyme activity, nutrient release, and chemical structure changes. Nitrogen or microbes may facilitate decomposition through oxidase or hydrolase activity improvement, while nitrogen-combined microbes increased all kinds of enzyme activity, causing a higher availability of nutrients to be released and a larger decomposition potentiality of the straw compost. The integration of a nitrogen fertilizer and a straw-decomposing microbial inoculant not only stimulated straw decomposition and enzyme activity better than a sole submission but also exhibited a lesser decay level, which includes the potential for further straw decomposition after 120 days.

## Figures and Tables

**Table 1:** Temporal change in relative abundances of functional groups (%) and in straw obtained by multi CP-NMR technique and A/OA ratio, aromaticity index, and hydrophobicity index under different conditions.

Time	Treatment	220–188 ppm	188–162 ppm	162–142 ppm	142–113 ppm	113–93 ppm	93–64 ppm	64–44 ppm	44–0 ppm	Other		
Days	Applied	C=O	COO/N-C=O	Aromatic C-O	Aromatic C-C	Anomeric C	O-Alkyl C	OCH3	Alkyl C	A/OA	Aromaticity	Hydrophobic
7	CK	NA	1.10 ± 0.26 c	2.42 ± 0.13 c	4.04 ± 0.31 c	16.2 ± 0.14 a	55.6 ± 0.35 a	15.2 ± 0.46 a	6.10 ± 0.24 a	0.11 ± 0.00 a	0.06 ± 0.00 c	0.23 ± 0.01 c
	NS	0.14 ± 0.24 ab	2.48 ± 0.36 b	3.53 ± 0.07 b	6.19 ± 0.35 b	16.7 ± 0.27 a	53.3 ± 0.62 b	12.7 ± 0.68 b	4.96 ± 0.68 a	0.09 ± 0.01 a	0.10 ± 0.01 b	0.27 ± 0.02 bc
	MS	0.09 ± 0.16 ab	2.41 ± 0.24 b	3.39 ± 0.07 b	6.01 ± 0.32 b	16.6 ± 0.7 a	53.3 ± 1.77 b	13.2 ± 0.75 b	5.07 ± 1.54 a	0.10 ± 0.03 a	0.01 ± 0.01 b	0.27 ± 0.04 b
	NMS	0.34 ± 0.08 a	3.53 ± 0.43 a	4.23 ± 0.15 a	7.00 ± 0.39 a	16.2 ± 0.13 a	50.7 ± 1.10 c	12.5 ± 0.09 b	5.63 ± 0.29 a	0.11 ± 0.01 a	0.11 ± 0.01 a	0.30 ± 0.02 a
15	CK	1.94 ± 0.30 a	4.69 ± 0.61 a	4.27 ± 0.16 a	8.16 ± 0.27 a	14.7 ± 0.26 a	42.3 ± 1.45 a	14.5 ± 0.69 a	9.52 ± 1.14 a	0.23 ± 0.03 a	0.14 ± 0.00 a	0.50 ± 0.05 a
	NS	2.03 ± 0.36 a	5.02 ± 0.22 a	4.34 ± 0.14 a	7.90 ± 0.89 a	14.5 ± 0.30 a	41.7 ± 2.06 a	14.8 ± 0.70 a	9.69 ± 1.36 a	0.23 ± 0.04 a	0.13 ± 0.02 a	0.50 ± 0.07 a
	MS	1.64 ± 0.51 a	4.81 ± 0.57 a	4.17 ± 0.44 a	7.62 ± 0.50 a	14.3 ± 0.21 a	41.0 ± 1.51 a	15.5 ± 1.39 a	11.1 ± 1.65 a	0.24 ± 0.05 a	0.13 ± 0.01 a	0.41 ± 0.05 a
	NMS	1.84 ± 0.17 a	4.96 ± 0.39 a	4.53 ± 0.02 a	7.68 ± 0.07 a	14.3 ± 0.31 a	41.5 ± 0.91 a	15.3 ± 0.35 a	9.84 ± 0.69 a	0.27 ± 0.02 a	0.13 ± 0.00 a	0.51 ± 0.02 a
30	CK	1.85 ± 0.24 a	4.53 ± 0.28 a	4.36 ± 0.03 a	8.16 ± 0.50 a	14.6 ± 0.12 a	41.3 ± 1.50 a	14.9 ± 0.36 a	10.3 ± 1.47 a	0.25 ± 0.04 a	0.14 ± 0.01 a	0.53 ± 0.07 a
	NS	2.02 ± 0.67 a	4.97 ± 1.29 a	4.58 ± 0.54 a	8.01 ± 0.81 a	14.4 ± 0.64 a	40.7 ± 2.88 a	15.2 ± 0.75 a	10.1 ± 2.01 a	0.25 ± 0.06 a	0.14 ± 0.02 a	0.54 ± 0.08 a
	MS	2.09 ± 0.73 a	5.38 ± 0.83 a	4.89 ± 0.28 a	8.49 ± 0.29 a	14.3 ± 0.11 a	39.9 ± 0.31 a	14.4 ± 0.74 a	10.6 ± 1.24 a	0.26 ± 0.03 a	0.14 ± 0.01 a	0.57 ± 0.02 a
	NMS	1.98 ± 0.19 a	5.29 ± 0.60 a	4.62 ± 0.15 a	8.08 ± 0.22 a	13.8 ± 0.78 a	39.6 ± 2.28 a	15.0 ± 0.21 a	11.7 ± 2.12 a	0.30 ± 0.07 a	0.14 ± 0.00 a	0.59 ± 0.09 a
70	CK	2.35 ± 1.03 a	6.20 ± 1.06 ab	4.85 ± 0.45 ab	9.66 ± 0.91 a	12.2 ± 0.42 b	31.1 ± 1.81 b	16.1 ± 1.44 a	17.5 ± 1.05 a	0.57 ± 0.05 a	0.17 ± 0.02 a	0.96 ± 0.08 a
	NS	1.94 ± 0.42 a	5.13 ± 1.22 b	4.57 ± 0.22 b	8.26 ± 0.92 a	13.9 ± 1.03 a	39.6 ± 4.74 a	15.3 ± 0.10 a	11.4 ± 3.07 b	0.30 ± 0.12 b	0.14 ± 0.02 a	0.59 ± 0.17 b
	MS	2.36 ± 0.15 a	7.00 ± 0.29 a	5.27 ± 0.06 a	9.16 ± 0.39 a	12.0 ± 0.43 b	31.9 ± 1.27 b	16.3 ± 0.24 a	16.0 ± 1.06 a	0.50 ± 0.05 a	0.16 ± 0.01 a	0.89 ± 0.07 a
	NMS	2.24 ± 0.24 a	6.12 ± 0.44 ab	4.78 ± 0.13 ab	9.33 ± 1.63 a	12.7 ± 0.55 ab	33.6 ± 3.79 ab	15.8 ± 0.60 a	15.5 ± 2.52 a	0.47 ± 0.13 ab	0.16 ± 0.03 a	0.84 ± 0.20 ab
120	CK	2.84 ± 0.71 a	6.58 ± 0.47 a	5.59 ± 0.51 a	10.3 ± 0.95 a	13.5 ± 0.58 a	33.4 ± 0.87 b	14.2 ± 1.19 a	13.5 ± 1.45 a	0.40 ± 0.04 a	0.18 ± 0.02 a	0.81 ± 0.01 ab
	NS	3.27 ± 0.21 a	6.63 ± 0.06 a	5.47 ± 0.10 a	10.9 ± 0.25 a	13.1 ± 0.13 a	32.2 ± 0.41 b	14.2 ± 0.16 a	14.3 ± 0.43 a	0.44 ± 0.02 a	0.19 ± 0.00 a	0.87 ± 0.02 ab
	MS	2.90 ± 0.37 a	5.78 ± 1.08 ab	5.58 ± 0.98 a	10.0 ± 1.31 a	13.2 ± 0.77 a	31.9 ± 1.70 b	15.8 ± 1.60 a	14.9 ± 2.29 a	0.47 ± 0.09 a	0.18 ± 0.03 a	0.88 ± 0.10 a
	NMS	1.71 ± 0.54 b	5.00 ± 0.38 b	4.76 ± 0.09 a	8.03 ± 0.03 b	13.0 ± 0.48 a	36.1 ± 1.80 a	16.5 ± 1.14 a	15.0 ± 1.45 a	0.42 ± 0.06 a	0.14 ± 0.00 b	0.74 ± 0.08 b

Note: CK, straw natural composting; NS, straw compositing with nitrogen application; MS, straw com-positing with SDMI application; NMS, straw compositing with nitrogen and SDMI co-application. CK, straw natural composting; NS, straw compositing with nitrogen application; MS, straw com-positing with SDMI application; NMS, straw compositing with nitrogen and SDMI co-application. Each value in table is the mean SD of three replicates. Numbers followed by different lowercase letters in the same column are significantly different at *p* < 0.05.

## Data Availability

The data presented in this study are available on request from the corresponding author.
